# Germ cell recovery, cryopreservation and transplantation in the California white sturgeon, *Acipenser transmontanus*

**DOI:** 10.1038/s41598-023-44079-6

**Published:** 2023-10-06

**Authors:** Amie L. T. Romney, Danielle M. Myers, Fatima R. Martin, Tawny N. Scanlan, Stuart A. Meyers

**Affiliations:** 1 Department of Anatomy, Physiology, and Cell Biology, School of Veterinary Medicine, University of California, Davis, CA USA; 2 Department of Biology, Center for Life in Extreme Environments, Portland State University, Portland, OR USA

**Keywords:** Animal biotechnology, Cellular imaging, Cell proliferation

## Abstract

The white sturgeon (*Acipenser transmontanus*) is the largest freshwater fish in North America. Because of the unique life history characteristics of sturgeon, including longevity, late maturation and long spawning intervals, their aquaculture can be a significant investment of resources. As a result of habitat loss and overharvesting, natural populations of white sturgeon are threatened and there is a growing effort to improve conservation aquaculture programs. Germ cell transplantation is an innovative technology previously demonstrated in a variety of fish species to be able to produce a surrogate broodstock. The technique relies upon optimal donor germ cell recovery and transplantation into a recipient fish. In this study, we developed and optimized the harvest of donor cells for germline transplantation and evaluated methods for ovary cryopreservation for the first time in the white sturgeon. We found that harvesting gonads from juveniles between the ages of 1.5 and 2.5-years resulted in reliably high proportions of pre-meiotic cells regardless of sex, a critical feature for using white sturgeon for transplantation studies since the species shows no distinguishing external sex characteristics. From the viable cells, we identified germline cells using immunolabeling with the antibody DDX4, a marker specific to the germline. For in vivo tracking of donor cells during transplantations, gonadal cells were stained with a long half-life non-toxic cell membrane dye, PKH26, and microinjected into the peritoneal cavity of newly hatched white sturgeon larvae. Larvae were reared until 3 months post-transplantation to monitor for colonization and proliferation of PKH26-labeled cells within the recipient larval gonads. Furthermore, viable cell detection, assessment of germline-specificity, and transplantation was determined for cells recovered from cryopreserved ovarian tissue from sexually immature females. Transplantations using cells cryopreserved with media supplemented with dimethyl sulfoxide (DMSO) rather than ethylene glycol (EG) demonstrated the highest number of PKH26-labeled cells distributed along the gonadal ridges of the larval recipient. Determining optimal methods of tissue cryopreservation, and germ cell recovery and transplantation are foundational to the future development of germ cell transplantation as a strategy to improve the aquaculture and conservation of this species. Our study demonstrates that conservation actions, such as surrogate breeding, could be utilized by hatcheries to retain or improve natural gamete production without genetic modification, and provide an encouraging approach to the management of threatened sturgeon species.

## Introduction

Ancient giants, sturgeons have survived relatively unchanged from their origination 245 million years ago^1^. Yet in recent decades, Acipenseriformes have been assessed as the animal order most at risk of extinction on the planet^2^. All species of sturgeon in this order are classified by the International Union for Conservation of Nature (IUCN) as Vulnerable, Endangered, or as Critically Endangered: the ultimate category of a threatened species prior to extinction^2^. The largest North American sturgeon, the white sturgeon (*Acipenser transmontanus*), inhabits coastal regions and large river systems along the West Coast of Canada and the United States. Anthropogenic impacts on white sturgeon such as overharvesting, pollution, habitat alteration and dam construction has sparked an increase in recovery efforts and research for the conservation of this species^3^. The large commercial production of sturgeon in North America^4–6^—a result of decades of research on sturgeon reproductive biology and embryology—positions white sturgeon as an ideal model for developing advanced breeding technologies for conservation aquaculture initiatives^7–10^.

Previous conservation aquaculture programs were based on collecting gametes from annual captures of reproductively mature wild white sturgeon with subsequent fertilization at a hatchery^11^. However, these strategies have limitations with hatchery-practiced annual spawning events given the rare availability and low genetic diversity. One practical approach to enhance fish production is surrogacy, in which germline chimeras are used to produce donor-derived gametes. Surrogate production comprises two critical techniques: (1) isolating gamete precursors and (2) transplanting stem cells into recipients (surrogates), either of the same or a related species^12–16^. There are key advantages to using early-stage germ cells—such as primordial germ cells and early stages of oogonia or spermatogonia—as donor cells. First, they retain sexual bipotency and have the ability to differentiate into either egg or sperm, depending on the sex of the recipient^17,18^. Additionally, transplanted donor cells are similar to endogenous germline stem cells in that they have the ability to migrate to and colonize the genital ridges of recipient fish^19,20^. Lastly, a single germline stem-cell can be transplanted into larval recipient gonads for generating adult germline chimeras across related populations and even related species for large-scale surrogate production^13,21^. For small broodstock populations that can be easily sustained in captivity (like that of white sturgeon), the focus should be on collecting early-stage or primordial germ cells from donor gonadal tissue rather than mature gametes: successful transplants require much fewer founder cells for generating successful germline chimeras that can be spawned year after year^22^. Surrogate broodstock technologies using germ cell transplantation have been developed in Russia, Japan and China for sturgeon and other fish species for commercial and conservation applications^16,23–28^. Outcomes of those studies have revealed that transplantation of germline stem cells can be successful for generating viable offspring from donor germline stem cells^15^.

Practical techniques such as donor selection, isolation of viable germ cells, along with labeling and transplanting those cells into a larval surrogate must be established prior to the optimization of surrogate production. It is important to consider the age at which to harvest gonadal tissue from a donor in order to obtain the maximum yield of immature oogonia or spermatogonia. Some of the challenges to using sturgeon as donors include the lack of sexually dimorphic external traits and the asynchronous sexual maturation in fish of similar age^29^. High variability in ovarian development has been observed in white sturgeon of the same age on commercial farms, likely in result of competition or density related factors^7^. Challenges are also presented with using sturgeon as larval recipients; there is a narrow time window of availability from managed broodstocks with spawning occurring annually^7^. However, if these challenges can be overcome, then methods of germ cell transplantation in the white sturgeon can be adapted to recover the segmented and genetically distinct white sturgeon populations and for related sturgeon species. In this study, we describe the development of a successful intraperitoneal germline stem cell transplantation strategy for the white sturgeon. This includes techniques of collecting gonadal tissue from young sexually differentiated sturgeon, the identification of germline stem cells, and allogeneic transplantations into newly hatched larval recipients.

## Results

**Figure 1:**
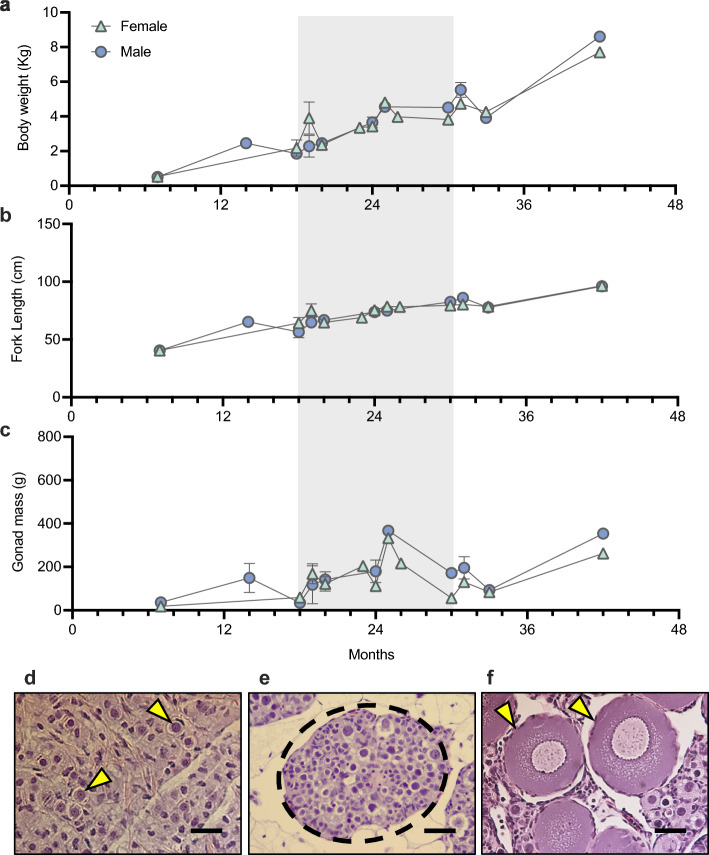
Characterization of juvenile white sturgeon *A. transmontanus* to determine the optimal donor age. Biometric parameters (mean ± SEM) including (**a**) body weight, (**b**) fork length, and (**c**) gonad mass. From these findings, the preferred age of donor gonadal tissue is 18–30 months and is marked in grey in (**a**, **b**, **c**). Histological examination of (**d**) 18 month-old testis shows populations of spermatogonia (yellow arrows) and Sertoli cells with no observable mature sperm remained; a consistent finding with all ages of males. Histological examination at 18 month-old female (**e**) shows nests of oogonia (dashed circle), however, at 36 months (**f**), the population of primary oocytes (yellow arrows) is more abundant. Scale bars for (**d**, **e**, **f**) are at the bottom right of each image and represent 20 µm, 50 µm, and 50 µm, respectively.

A survey of biometric parameters was performed on 73 juvenile white sturgeon (31 males and 42 females) across an age range of 7 to 42 months to investigate the optimal age range for germline stem cell recovery. Early juvenile sturgeon demonstrated increased body mass and fork length with age for males and females (Fig. 1a,b). The minimum body mass for males/females examined was 0.50/0.53 kg and the maximum was 8.6/7.7 kg, respectively. The minimum fork length examined for both sexes was 40.5 cm and the maximum fork length for males and females was 71.3 and 76.6 cm, respectively. No external features distinguishing males or females were detected. All fish were dissected to examine the gonads for maturity and sex. Three 7-month-old fish were examined but the developing gonads were likely too young and indeterminable as testis or ovary and therefore were not included. The gonads of both males and females were largely comprised of adipose tissue with a linear band of germinal tissue that can be used to distinguish testis from ovary. Measurements of gonadal mass increased with age from 34.2/18.0 g to 366.7/333.1 g for males/females (Fig. 1c). Upon histological examination, the germinal tissue in males at all ages had consistent profiles of spermatogonia throughout (Fig. 1d). Ovarian germinal tissue in females as young as 18 months-old contained large clusters of oogonial nests with few detectable oocytes (Fig. 1e). However, this pattern varied among individuals regardless of age, with higher numbers of primary oocytes detected (a signature of the onset of mitosis) as early as 36 months (Fig. 1f). Therefore, a narrow window of 18–30 months was selected as an optimal donor age for our purposes, since it would yield a large proportion of early germline stem cells regardless of sex.

For donor cell preparations, sections of germinal tissue in Leibovitz L-15 medium (pH 7.8; Life Technologies, Carlsbad, CA, USA) were washed in phosphate buffered saline (PBS), minced, and enzymatically dissociated with 0.3% trypsin. These steps allowed for a substantial yield of viable cells that could be used for experiments. In males (*n* = 21), 1 g provided an average of 3.3 × 10^6^ ± 4.1 × 10^5^ cells. In females (*n* = 15), 1 g resulted significantly more with an average of 9.6 × 10^6^ ± 1.8 × 10^6^ cells (t-test, *p* = 0.0003).

**Figure 2:**
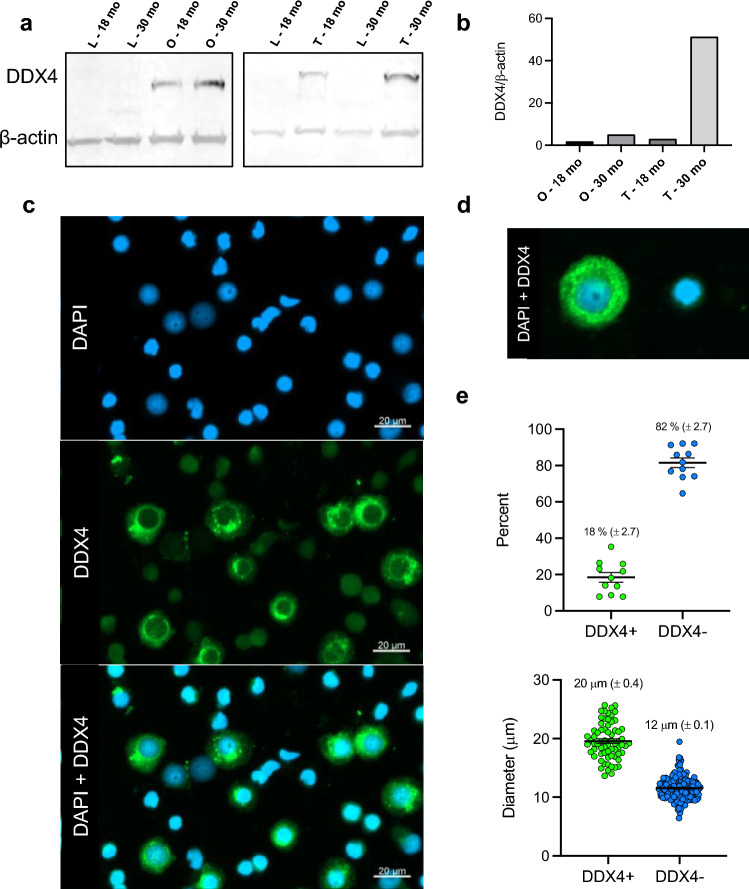
Germline identification using DDX4 antibody. (**a**) Western blot analysis of germ cell marker, DEAD-box helicase 4 (DDX4) compared to the eukaryotic-cell control β-actin detected in total protein extracted from juvenile sturgeon liver and gonad tissues (L = liver, O = ovary, and T = testis) from female (left) and male (right) specimens. (**b**) shows the comparison of DDX4 intensities normalized to β-actin intensity across the samples. A cropped blot is shown. A full-length blot is presented in Supplementary Fig. S1 online. (**c**) After dissociation, cells were fixed and stained with a primary antibody against DDX4 (secondary antibody against rabbit IgG) and counterstained with DAPI, a cell nuclear stain. (**d**) The resultant cell populations of DDX4-positive cells (DDX4+) were identified from DDX4-negative (DDX4-) cells, and counted under fluorescence microscopy (**e**, top). Under differential interference contrast (DIC), cell diameters were measured (**e**, bottom; µM, ± SEM). Under DIC, DDX4+ cells were statistically larger than DDX4- cells (t-test, *p* < 0.0001).

Using Western-blot analysis, the germline specific DDX4 antibody labeled a protein band at approximately 75 kDa in ovarian and testicular samples with no detection of the DDX4 antibody in liver tissue used as a negative control (Fig. 2a). After normalizing protein levels DDX4 to that of β-actin, we observed an increase in the presence of DDX4 determined by densitometric analysis in gonads at 30-months compared to gonads at 18-months (Fig. 2b; Supplementary Fig. S1), however, due to a lack of replicates for this analysis, significant results cannot be concluded.

Immunocytochemistry of cell suspensions from dissociated gonads (*n* = 8) showed germline specificity with DDX4-labeled (DDX4+) cells detected amongst cells without DDX4 labeling (DDX4−; Fig. 2c). Once germline cells were confirmed with localization of the antibody in the perinuclear space (Fig. 2d), we counted and measured the two populations of cells. The DDX4+ cells (confirmed germline) contributed to an average of 18 ± 2.7% of the cell population across multiple individuals (Fig. 2e, top). Under differential interference contrast (DIC) microscopy, DDX4+ cells were statistically larger (20 ± 0.4 µm) than DDX4− cells (12 ± 0.1 µm) in diameter (*n* = 183 cells measured from 8 individuals, t-test, *p* < 0.0001; Fig. 2e, bottom).

**Figure 3:**
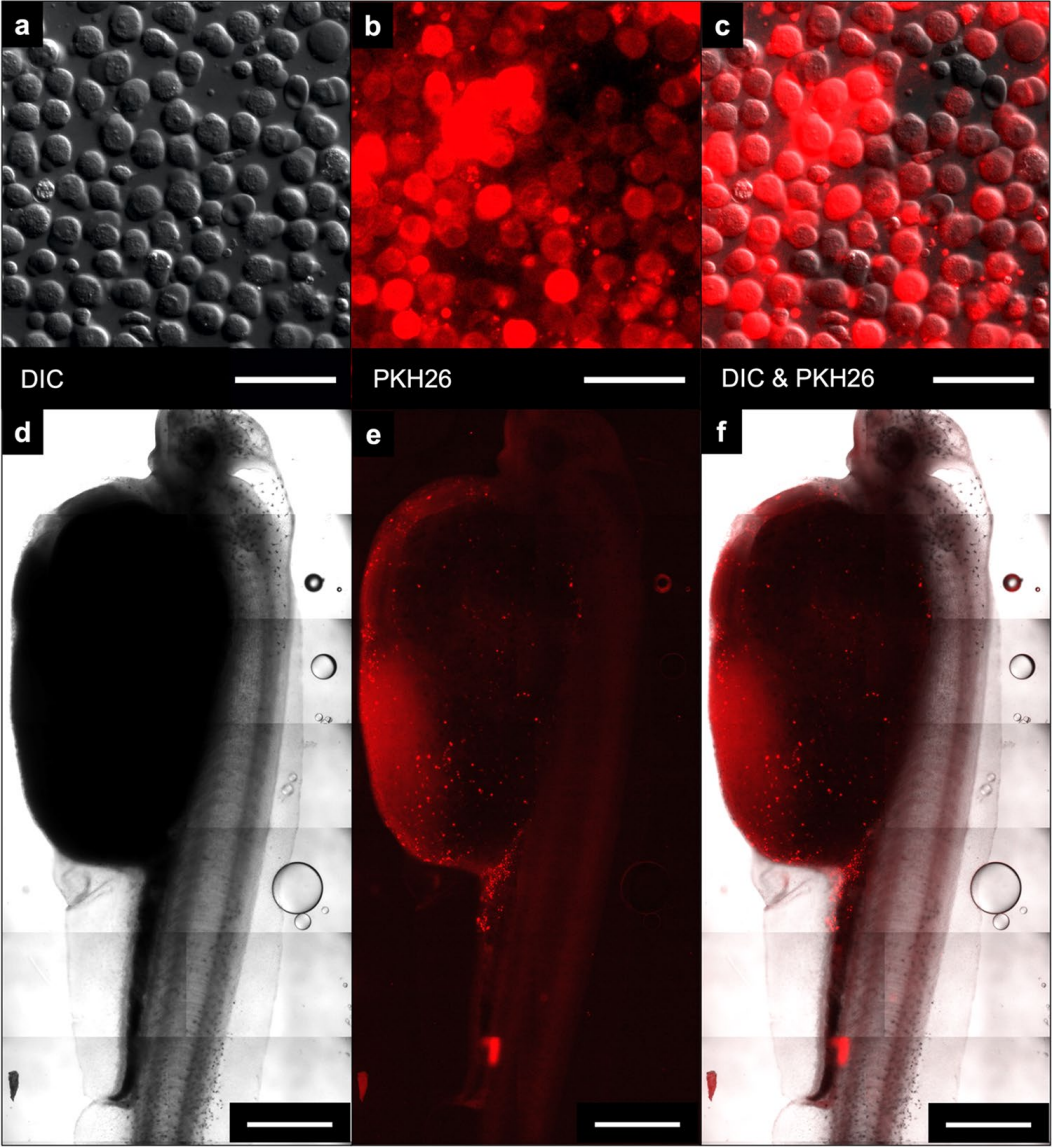
PKH26-labeled cells for in vivo tracking of germ cell transplantation. Gonadal cells harvested white sturgeon ovaries and labeled with the red fluorescence dye PKH26 prior to transplantation. Image (**a**) shows cells under differential interference contrast (DIC) and red fluorescence was detected with fluorescence microscopy (**b**) prior to injections. Intracellular localization of red fluorescence can be seen in merged image (**c**). Tiled images of white sturgeon larvae imaged under DIC (**d**) and under fluorescence for detection of PKH26-labeled cells (**e**). The merged image (**f**) shows the intraperitoneal localization of the labeled cells within the larvae. Images (**d**, **e**, **f**) were taken immediately after performing injections. Scale bars are located at the bottom right corner of each image; for (**a**, **b**, **c**) = 50 μm and for (**d**, **e**, **f**) = 1 mm. Red fluorescence was detected with excitation/emission wavelength = 597/624 nm.

We improved the cell dye PKH26 (Sigma-Aldrich) staining for *A. transmontanus* by increasing the concentration recommended in the manufacturers protocol by two times, resulting in fluorescently stained cell populations that could be efficiently observed over three months (Fig. 3a–c). For the duration of transplantation experiments we used female-sourced cells stained with PKH26 for in-vivo tracking within recipient larvae. From cell density calculations, injections consisted of 5000 to 50,000 cells across volume of 0.05 to 0.2 µl per fish.

**Figure 4:**
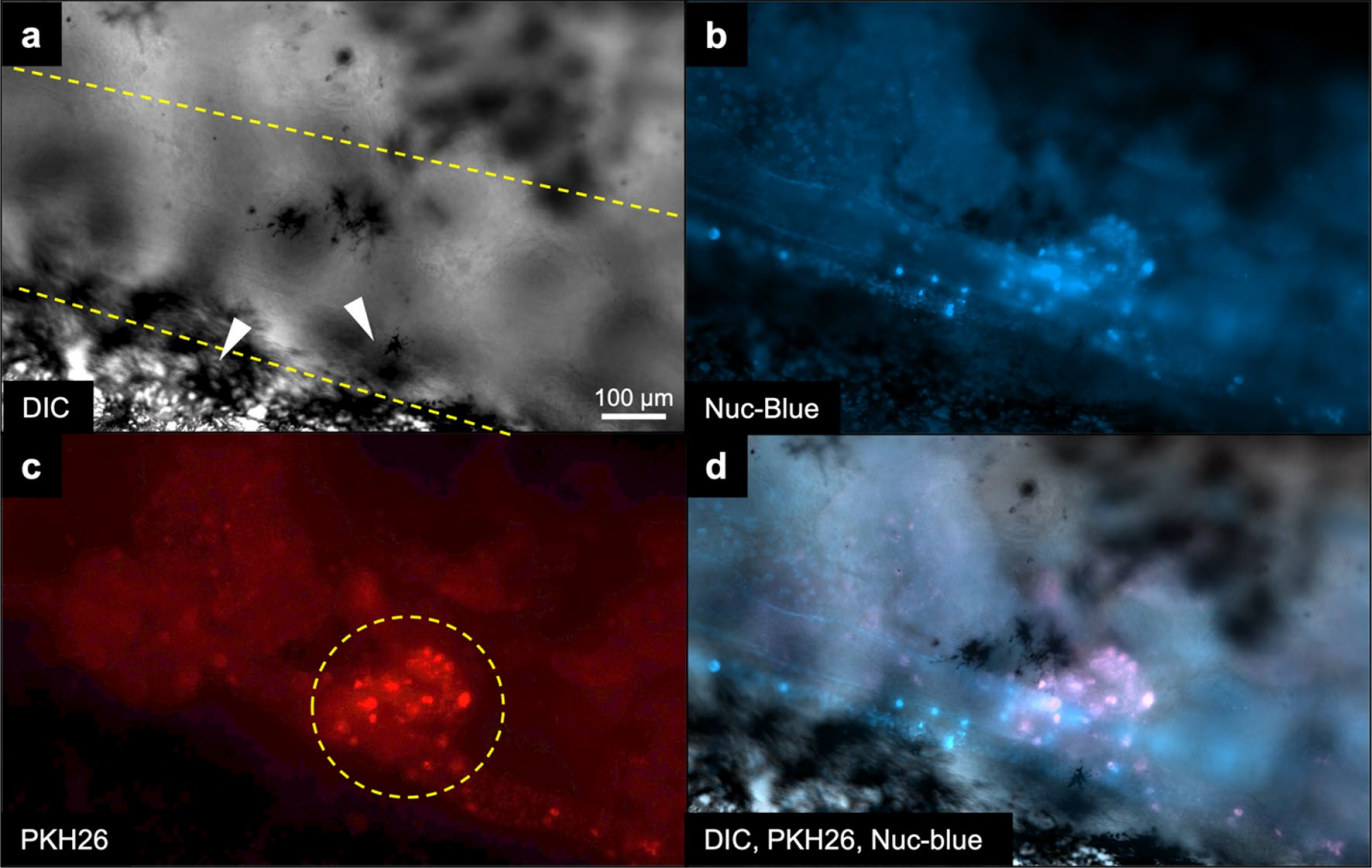
PKH26-labeled white sturgeon stem cells injected into larval recipients counterstained internally with blue nuclear stain. (**a**) Larval recipients imaged under differential interference contrast (DIC) show the developing gonadal ridge demarcated with dashed yellow lines where epithelial cells transition in and out of focus (white arrowheads). (**b**) Incubation of abdominal tissue with the nuclear stain NucBlue shows cellular structure of the gonadal ridge. (**c**) PKH26-labeled cells were detected along the developing gonadal ridge and fold; an example here shows a cluster of punctate red signals (yellow dashed line circle). (**d**) Overlapping the images aligns the transplanted cells with the developing gonadal ridge. In all images, the axis of the body is at an angle with the anterior toward the bottom right and posterior to the top left. The scale bar for all images is located in (a) and represents 100 µm.

To reduce the handling time of fish during the experiments, most fish were transferred directly back to flow tanks for recovery after undergoing transplantations. However, a few larvae were examined under magnification immediately post-injection to confirm microinjection-delivery of labeled germline cells (Fig. 3d–f). PKH26-labeled cells were visualized circulating around the yolk and throughout the peritoneal cavity. A total of 385 larval white sturgeon (0-, 1- or 2-days post-hatch) acquired across three spawning events were used as recipients of intraperitoneal injections of PKH26-labeled donor cells. Of those recipient larvae, 67% survived from the period of injection to the yolk absorption and transition to feed. After three months, 146 recipients were dissected and examined for the presence of fluorescently labeled cells within the bilateral gonadal ridges along the dorsal abdominal wall. In 112 out of those dissected (76.7%), fluorescence signal was detected within the region of the developing gonadal ridge. In control fish (*n* = 12) that did not receive injections with PKH26-labeled cells, no fluorescence was detected. The PKH26-stained (transplanted) cells were detected as red punctate signals bilateral to the body midline in contrast to surface cells detected by incubation of the abdominal trunk tissue in a nuclear stain, Nuc-Blue, immediately after dissection and prior to imaging (Fig. 4a–d).

**Figure 5:**
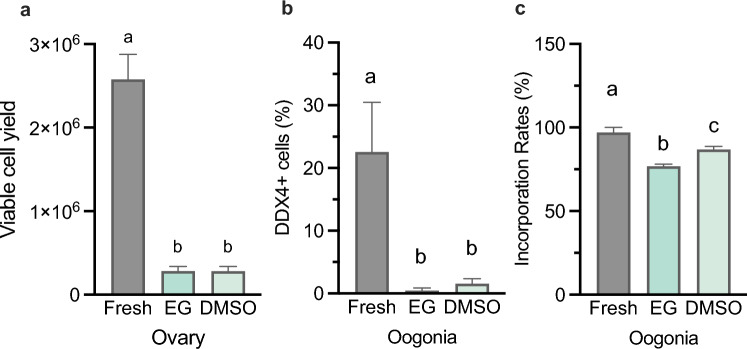
Cell recovery outcomes from tissue cryopreservation and use in transplantations. Outcomes of (**a**) viable cell yield from fresh and post-thawed ovarian sections when cryopreserved with media supplemented with ethylene glycol (EG) or dimethyl sulfoxide (DMSO). (**b**) The percent of cells labeled with DDX4 (DDX4+) recovered from fresh and cryopreserved ovarian tissue using cryomedia with EG or DMSO. (**c**) The percent of larvae recipients at three months after transplantations with fluorescently tagged cells incorporated along the gonadal ridge. Letters above each bar represent the statistical similar groups determined by ANOVA, p < 0.05).

Two supplements, ethylene glycol (EG) and dimethyl sulfoxide (DMSO), were used to optimize tissue cryopreservation on ovarian sections from individuals of *A. transmontanus*. The effectiveness of cryopreservation components was assessed by comparing total viable cells recovered per unit mass of tissue and the proportion of DDX4+ cells that were detected after three months of tissue storage in liquid nitrogen. These values were compared to tissues from the same individuals that did not undergo cryopreservation. No statistical difference in viable cell yield was detected after cryopreservation with cryomedium supplemented with EG or DMSO yet both cryopreservation treatments resulted in significantly lower numbers of viable cells compared to that of freshly dissociated tissue (ANOVA, *p* = 0.0028) (Fig. 5a). Further, both cryopreservation methods resulted in significantly lower numbers of DDX4+ cells (ANOVA, *p* = 0.0248) but were not different from each other with regard to EG or DMSO (Fig. 5b). Additional ovarian samples from the same individuals were dissociated after thaw and labeled with PKH26 for transplantation comparisons of cryomedia. Interestingly, ovarian tissue cryopreserved using DMSO resulted in higher successful transplantation outcomes compared to that of EG (ANOVA, *p* = 0.0105) (Fig. 5c).

## Discussion

Germ cell transplantation technology has in the last two decades been developed as a promising assistive reproductive technology for the conservation and cultivation of fish that are commercially relevant or threatened^30^. Here, we develop and test the isolation and cryopreservation methods that are necessary to establish germ cell transplantation for the white sturgeon, a species that is both threatened and commercially important. Specifically, we have overcome two technical challenges associated with using white sturgeon: (1) the characterization and optimal recovery of germ cells from fresh and cryopreserved donor tissue and (2) the transplantation of membrane-labeled gonadal cells and tracking their incorporation into recipient gonads.

We found that in captively bred juvenile *A. transmontanus* specimens, male and female gonads are distinguishable anatomically and cytologically as early as 18 months of age, despite undetectable external sexual dimorphism, which is similar to the findings reported by Doroshov et al.^7^. We observed gonadal maturation in *A. transmontanus* that follows the staging of Siberian sturgeon, *A. baeri*^31^. It was important to determine an age range specific to this species that would suffice for sourcing early-stage germ cells without needing to know the sex of the gamete donor. An added advantage associated with this procedure is that germ cells have the potential to differentiate into eggs or sperm, and it depends on the sex of the recipient. This work allows for future development for the generation of germ cell transplantation technology in the white sturgeon that would utilize surgically biopsied tissue from donor individuals rather than euthanasia and sacrifice of genetically valuable individuals. This would be especially useful for instances when donor tissue is limited in availability or accessibility.

Our work uncovered that small amounts of juvenile *A. transmontanus* gonadal tissue can be a rich source of cells with as little as 1 g generating millions of cells in donors of 18–30 months of age. Determining the presence of germline specificity in cells recovered from dissociated gonads is critical for the generation of germline chimeras in the surrogates. Similar to other species, germline cells in the white sturgeon are larger in diameter than surrounding somatic cells^32,33^. Despite our efforts to filter cell suspensions, a diverse population of somatic cells were included in our injections along with germline cells. Recent studies have successfully demonstrated that a single primordial germ cell has the capacity to colonize, and amplify cell numbers, completely replacing the germ line of a sterile recipient teleost^22^. Additionally, even though DDX4 is a definitive germline marker, it cannot be used to distinguish primordial germ cells from later stage cells such as primary growth oocytes^34,35^. A future step in our work to overcome this uncertainty would be to include alternate markers to distinguish the age class of germline cells used for transplants.

As shown in Figs. 3 and 4, we were able to label cells with PKH26 and track them following injections into recipient gonads and after three months of growth. We likely encountered a reduction in fluorescence signal in cells from the time of injection until reexamined three months later due to cell division and a dilution of the stain, but this cannot be confirmed. Examinations of larval recipients showed examples of the pair of gonads or only one gonad with PKH26-labeled cells. Fish that were not injected showed no punctate signal from PKH26-labeled cells nor autofluorescence. It is important to consider that somatic cells or cell debris could also be stained with PKH26 dye and transplanted into the peritoneum of the larvae and could attach to the epithelium of the recipient gonadal ridge. We did not confirm that the incorporated and proliferated donor-derived PKH26-labeled cells within the recipient gonadal ridge were specifically DDX4+. However, it is not fully understood if fish donor-derived somatic cells colonize and replicate in the recipient gonad and contribute to form a niche that favors the colonization of germline cells, as demonstrated in mammals^36^. It is important that future applications of this technology utilize protocols to determine the cell types in the recipients and confirm germ line specificity.

In an additional experiment, we examined whether cryopreserved donor cells could also colonize recipient gonads. We adapted previous freezing protocols that demonstrated in the Siberian sturgeon *A. baerii*^37^. Our experiments examined the use of either DMSO or ethylene glycol as cryoprotectants during freezing. After thawing, ovarian tissue sections under either treatment resulted in a reduction of germ cell recovery; even so, we were able to successfully identify PKH26-labeled cells within larval gonads three months after transplantation. However, we cannot state with certainty that these cells were DDX4+. Yet it is encouraging in light of recent findings from other fish species supporting the possibility that low numbers of germline cells are required for successful colonization of recipient gonads^13,21^.

Future efforts essential for establishing germ cell transplantation in the white sturgeon would be the generation of sterile hosts (larval recipients). Functionally sterile fish can be mass produced in several fish species and supports the distinction of donor gamete production from the chance production of endogenous gametes^25,38^. Sterilization techniques in fishes is commonly carried out by triploidization or hybridization, yet because of being evolutionary polyploids, all ploidy levels in sturgeon have the probability of fertility^39^. Sterilization was achieved in the sterlet, *Acipenser ruthenus* by knock-out experiments on the germ cell exclusion-specific gene, *dead end*, or by depletion of primordial germ cells in embryos using ultraviolet irradiation^40–42^.

The white sturgeon is a robust candidate species for developing assisted reproductive technologies such as germ cell transplantation for the purpose of surrogate broodstock production. Efforts to preserve wild California white sturgeon populations include strategies such as annual monitoring, harvest restrictions, and habitat restoration. In addition to these, conservation aquaculture aims to enhance sturgeon population segments where natural recruitment (survival of larval fish to later life stages) is limited^11^. The species as a whole is federally listed as endangered under Canada’s Species at Risk Act^43^ and specific populations such as the Kootenai River white sturgeon is listed as endangered under the U. S. Endangered Species Act^44^. Sturgeon hatcheries are known to play an important role in the recovery plans for white sturgeon and have the potential to prevent further declines until habitat restoration can regain the recruitment losses that currently exist^45^. Captive breeding programs have been in place for decades to supplement wild populations yet contribute to serious consequences such as inbreeding and loss of genetic variation^46^. One recent study examined single-nucleotide polymorphisms among the segmented populations of white sturgeon throughout the Columbia River and adjacent basins and found little genetic diversity between groups, and further suggest that populations would benefit from controlled supplementation from proximal groups^47^. Even still, commercial production of white sturgeon has grown rapidly over the last few decades and has been successful in meat and caviar production^4^. The industry has successfully driven government supported research and development that has application to breeding programs and broodstock maintenance that can be applied to the conservation aquaculture efforts^11,48^.

In addition to improved production of white sturgeon, this technology is generally suitable for all fishes yet particularly for wild sturgeon species which are suffering globally from population declines. A close relative to *A. transmontanus* is the North American green sturgeon, *Acipenser medirostris*. Under the United States Endangered Species Act, the Southern distinct population segment of *A. medirostris* has been listed as Threatened due to reduced spawning habitats^48,49^. A captive population originated from the *A. medirostris* Northern segment with the assistance of the Yurok Tribe and has been maintained by the Green Sturgeon Broodstock program at the University of California Davis^50^. Broodstock technologies such as germ cell transplantation are well adapted for small breeding populations and would be a practical approach for the recovery of a threatened species, such as the green sturgeon.

Surrogate broodstock production using germline chimeras in result of germ cell transplantation can be used to expand white sturgeon conservation breeding programs and has the potential to improve broodstock maintenance. Whether future efforts are aimed toward the application of allogeneic (demonstrated here) or xenogeneic models using white sturgeon—perhaps incorporating related sturgeon species—the outcomes will facilitate improved fish production that would prove invaluable to aquaculture research. Determining optimal methods for germline detection and cryopreservation supports this new reproductive management tool for wild sturgeon recovery efforts, and for hatcheries to utilize natural gamete production without genetic modification.

## Methods

### Animal ethics

All animal care and experimental procedures were approved by the University of California Davis Institutional Animal Care and Use Committee. Methods of euthanasia for small fish (< 100 g) included an overdose of buffered tricaine methanesulfonate at 500 mg/L. For larger fish (> 1 kg), fish were manually applied blunt force trauma to the head followed by transection of the cervical spinal cord according to AVMA guidelines for the euthanasia of animals: 2020 edition^51^. Information provided in the manuscript complies with the essential recommendations for reporting of the ARRIVE guidelines.

### Recipient white sturgeon embryo and larval care

White sturgeon embryos were acquired immediately upon fertilization from multiple Californian farms throughout March and April 2020. Upon acquisition, populations of embryos (~ 2000 to 4000) were transported to CABA and held in aquaria equipped with McDonald- or upwelling-jars with incubation temperatures at 12 °C (± 1 °C) or 14 °C (± 1 °C) where they remained during embryogenesis. Embryos were monitored daily for developmental timing. Newly hatched larvae were collected into floating basket nurseries and collected upon initiation of transplantation experiments. During the experiments, larvae were maintained at 12 °C and temporarily immobilized using buffered tricaine methanesulfonate according to IACUC policy “Guidelines for Euthanasia of Research and Teaching Animals”. After transplantation experiments were conducted, larvae were returned to floating nurseries in flow-through tanks for recovery and slowly acclimated to 18 °C (+ 1 °C per day). Prior to the loss of their yolk plug, larvae were fed artemia nauplii and transitioned to Rangen RSM Starter dried food (www.rangen.com; 55% protein, 17% fat, < 2% fiber, < 10% ash) once dropping the yolk plug. Larvae were reared until three months of age before being euthanized with buffered tricaine methanesulfonate and dissected to examine developing gonadal ridges for positively stained cells using a Zeiss Axio Imager microscope (Zeiss, Germany).

### Donor fish

Juvenile white sturgeon were acquired from Sterling Caviar LLC (Elverta, CA, U.S.A) and transported to outdoor 6-foot diameter flow-through tanks at the Center for Aquatic Biology and Aquaculture at the University of California, Davis, CA, U.S.A. Fish were held in densities at no more than 20 per tank and with temperatures at 18 ± 1 °C with exposure to a natural photoperiod. Fish were fed 5.0 mm sinking trout pellets (40% protein, 12% fat, 3% fiber, 12% ash; Skretting, Tooele, USA) at a feeding rate at 0.4 to 0.5% of total tank biomass.

### Donor gonad characterization

Donor sturgeon between 1 and 4 years in age were used to characterize gonadal tissue to determine the optimal age for isolating early germline stem cells such as oogonia and spermatogonia. Following euthanasia, body weight and fork length measurements were collected for comparisons of body growth and sexual maturation. Through an abdominal incision, gonads were isolated from viscera and weighed to determine total gonadal mass. Tissue fragments (1 cm^3^) of gonads were collected and fixed in 4% paraformaldehyde. Each sample was embedded in paraffin, sectioned, and stained with hematoxylin and eosin by the Anatomic Pathology Service at the University of California Davis Veterinary Medical Teaching Hospital. Images of tissue sections were obtained using brightfield microscopy at 200 × total magnification with a Zeiss Axio Imager microscope (Zeiss, Germany). Germ cell populations were identified in gonadal tissue sections according to the description by Lacerda et al.^52^.

### Gonad dissociation

Ovarian and testicular tissue was dissociated into a cell suspension following modified methods used previously for *A. baerii*^26^. Briefly, germinal portions of the gonads were dissected away from adipose tissue and transferred to L-15 for 1–2 h on ice. Germinal tissue was sectioned (0.5 ± 0.05 g), rinsed with phosphate buffered saline (PBS) adjusted to be isotonic to the blood plasma of sturgeon at 238 mOsm/kg and pH of 8, then minced into fragments and transferred into PBS supplemented with 0.3% trypsin and incubated for 2 h at 22 ± 1 °C on an orbital rocker for tissue digestion. Cellular digestion was stopped by diluting the samples by 50% (vol/vol) with PBS and 0.1% DNase. The obtained homogenate was passed through a 50 µm nylon filter (Partec, Germany), for isolating cells and nuclei from cell debris and aggregates. Filtered gonadal cell suspensions were rinsed with PBS and centrifuged to remove residual lipid (500×*g* for 30 min at 10 °C). Immediately after dissociation, subsamples of cells were stained with trypan blue to determine cell viability. The total number of cells (cell yield) and the proportion of live to dead cells (% viability) was determined using a hemocytometer and used to calculate viable cell yield.

### Protein extraction and SDS-PAGE

Total protein was extracted from sections (0.1 ± 0.05 g) of liver and gonads from juvenile white sturgeon at the ages of 18- and 30-months. Tissue sections were homogenized with a handheld homogenizer in a urea lysis buffer (8 M urea, 2 M thiourea, 4% CHAPS, 10% isopropanol, 0.1% Triton X-100, and 100 mM dithiothreitol) supplemented with Complete™ EDTA-free protease inhibitor mixture (Roche Applied Science). After a 2 h incubation at 4 °C, lysate was centrifuged at 12,000×*g* for 20 min and protein concentrations were measured with the Bradford protein assay^53^ using an Infinite M200 Pro plate reader (Männedorf, Switzerland) and default software settings (i-control software, Tecan). For SDS-PAGE, samples containing 20 µg were combined with Laemmli buffer and denatured at 85–90 °C for 10 min and then loaded to a 12% polyacrylamide gel for separation.

### Western blot

Protein was transferred to PVDF membranes for 30 min at 30 V. After electrotransfer, the membranes were washed with PBS, and nonspecific binding sites were blocked with 0.5% (wt/vol) nonfat dried skim milk and tris-buffered saline with the detergent 0.05% Tween-20 (25 mM Tris, 0.15 M NaCl, 0.05% Tween-20, pH 7.6) for 1 h. To examine expression profiles of the germ cell marker, DEAD-box helicase 4 (DDX4) from gonadal protein, membranes were incubated overnight at 4 °C with rabbit anti-DDX4 polyclonal antibody (1000-fold dilution; 0.5% nonfat dried skim milk-TBST; GTX116575, GeneTex, Inc.) as well as with anti-β-Actin Antibody (#4967S; Cell Signaling Technologies) for a loading control^54–59^. Membranes were rinsed and transferred to Alexa fluor 680 goat anti-rabbit immunoglobulin G secondary antibody (#A27042, Thermofisher) and images were captured with the Odyssey Infrared Imaging System (LI-COR, Lincoln, NE) imaging system. Quantification of immunoreactive bands using standard densitometric analysis was expressed as a ratio against β-actin using ImageJ software (NIH, Bethesda, MD) according to Davarinejad^60^. For full-length western blot analysis see Supplementary Fig. S1a and b.

### Immunocytochemistry

Immunostaining was performed initially on gonadal cell suspensions after dissociation to confirm the germ cells positively labeled for DDX4 (DDX4+) in cell suspensions based on a specific pixel intensity threshold. Ovarian cell suspensions were examined for proportions and sizes of DDX4+ cells per sample. Cell suspensions were fixed with 4% PFA at room temperature and then rinsed twice in PBS. Cells were transferred to poly-l lysine-coated slides by use of a Cytospin™ 4 Cytocentrifuge (ThermoScientific) and disposable Cytofunnel™ chambers. Spots of cells with 1 × 10^5^ to 1 × 10^6^ cells were created. Slides were briefly submerged into 4% PFA for a second fixation and to secure their adhesion to the slide before undergoing permeabilization with Triton X-100 (0.5%). After incubating in a blocking buffer consisting of 1% (wt/vol) bovine serum albumin in tris buffered saline with Tween-20, cells were stained with the primary antibody anti-DDX4 (same antibody as previous, 1:300) in blocking buffer at 4 °C overnight. The slides were washed and incubated with a secondary antibody, anti-rabbit immunoglobin G-fluorescein isothiocyanate (F0382, Sigma-Aldrich) for 1 h at room temperature. Lastly, cells were briefly incubated in the nuclear stain 4′, 6-diamidino-2-phenylindole (DAPI, 1%) before allowed to dry and mounted with VECTASHIELD® antifade mounting medium (H-1000-NB; Vector Laboratories, Peterborough, UK). Cells were imaged at 250 × total magnification under both differential interference contrast (DIC) and under fluorescence for analysis using a with Zeiss Axio Imager microscope and with ZEN blue software (Zeiss, Germany). DDX4+ and cells without the marker (DDX4−) were counted and measured under DIC.

### Staining and transplantation of gonadal cells

Microinjection needles were prepared ahead of time from thin-walled borosilicate glass capillaries (G-100, Narishige International, Inc, Tokyo, Japan) using pre-programmed settings (Heat: 710, Pull: 35, Velocity: 135, Time: 210) on a Puller model P-97 (Sutter Instruments, Novato, CA, USA). Needles were opened and beveled at an angle of 20° until the aperture width was between 55 and 60 µm on a Narishige microgrinder (EG-402, Narishige International, Inc, Tokyo, Japan). Cell densities within suspensions were determined by use of hemocytometer. For in-vivo tracking of cells during transplantations, gonadal cell suspensions from ovaries were labeled using the PKH26 Red Fluorescent Cell Linker Kit for general cell membrane labeling (PKH26GL, Sigma-Aldrich) with slight modifications to the manufacturer’s protocol (20 µM PKH26 for 10^7^ cells, incubated for 10 min in the dark). The recipient larvae were anesthetized with 0.001% buffered tricaine methanesulfonate and placed onto a 3% agarose mold with linear depressions for immobilization and surrounded with ice to reduce overheating. PKH26-labeled cells were loaded into a glass needle and injected into the peritoneal cavity posterior to the yolk sac and near the presumptive gonadal ridge using a Narishige oil microinjector (IM-21, Narishige International, Inc, Tokyo, Japan) under a Leica S9i Stereomicroscope (Leica Microsystems Co., Ltd., Weztlar, Germany). Fish that did not receive injections were maintained in a separate container alongside microinjected fish and identified as a control group. At the completion of transplantation experiments cells positively labeled with PKH26 were distinguished from cell debris by staining the eviscerated body cavity with NucBlue Live reagent (Hoechst 33,342 dye) in 75% ethanol to aid in penetration for 3 to 5 min to stain cellular nuclei in situ. Efficient transplantation of donor-derived germ cells was determined by the detection of red fluorescence puncta in recipient gonads.

### Cryopreservation of ovarian tissue

To evaluate cryopreservation techniques, we collected 0.25 g ovarian sections (*n* = 5 fish, 3 replicates per fish), and transferred the sections to cryotubes containing cryomedium consisting of isotonic PBS (238 mOsm kg^−1^). We followed cryomedia treatments from previous cryopreservation work in *A. baerii*^32^, using cryomedia treatments supplemented with either cryoprotectant ethylene glycol (EG) or dimethyl sulfoxide (DMSO), each at 1.5 M concentrations. Additional cryomedia components included bovine serum albumin (0.5%) and glucose (50 mM). Samples were equilibrated on ice for 10 min before being frozen at a rate of − 1 °C/min for 3 h using a BiCell freezing container (Nihon Freezer, Tokyo, Japan) placed at − 80 °C before being plunged into liquid nitrogen. After at least 2 months, cryotubes were thawed in a water bath at 10 °C for five minutes and rehydrated in a series of 3 × 5-min immersions in L-15 medium before undergoing cell dissociation. Viable cell yields and total number of DDX4+ cells were determined for each sample and treatment and then compared to values from fresh tissues for each fish. Cryopreserved samples and their corresponding fresh cell samples were stained with PKH26 and transplanted as described above and monitored three months later for colonization of PKH26-labeled cells in larval recipient gonads.

## Statistical analysis

All values are represented as mean ± standard error of the mean (SEM). Cell yields between males and females and DDX4 antibody detection were analyzed using Student's two-tailed t-test. Percentage data were tested for normality (Shapiro–Wilk test) and homogeneity of variance (plot of residuals vs. predicted values), before being tested by one-way ANOVA followed by Tukey’s HSD post-hoc test. Multiple comparisons after one-way ANOVA followed by Tukey’s HSD post-hoc test was used to examine the effects of different cryomedia recipes on post-thaw viable cells in compared to that of fresh tissue. All statistical analyses were conducted in GraphPad Prism version 9.4.1 (GraphPad Software, San Diego, CA).

### Supplementary Information

Supplementary Figure S1.

## Data Availability

The datasets generated during and/or analyzed during the current study which are not publicly available and referenced above are available from the corresponding author on reasonable request.

## References

[1] Gardiner BG, Eldredge N, Stanley SM (1984). Sturgeons as living fossils. Living Fossils.

[2] Crossman, J. & Hildebrand, L. *Acipenser transmontanus.* The IUCN Red List of Threatened Species 2022. e.T234A97440736 edn (2022).

[3] Ulaski ME, Blackburn SE, Jackson ZJ, Quist MC (2022). Management goals for conserving white sturgeon in the Sacramento-San Joaquin river basin. J. Fish Wildl. Manag..

[4] Bronzi P, Rosenthal H (2014). Present and future sturgeon and caviar production and marketing: A global market overview. J. Appl. Ichthyol..

[5] Ethier, V. Farmed sturgeon. in *Seafood Watch. Seafood Report*. (Monterey Bay Aquarium, 2014).

[6] Lee S, Sonmez O, Hung SS, Fadel JG (2017). Development of growth rate, body lipid, moisture, and energy models for white sturgeon (*Acipenser transmontanus*) fed at various feeding rates. Anim. Nutr..

[7] Doroshov SI, Moberg GP, Van Eenennaam JP (1997). Observations on the reproductive cycle of cultures white sturgeon, *Acipenser transmontanus*. Environ. Biol. Fish..

[8] Gille DA (2017). Finishing diet, genetics, and other culture conditions affect ovarian adiposity and caviar yield in cultured white sturgeon (*Acipenser transmontanus*). Aquaculture.

[9] Schreier A, Stephenson S, Rust P, Young S (2015). The case of the endangered Kootenai River white sturgeon (*Acipenser transmontanus*) highlights the importance of post-release genetic monitoring in captive and supportive breeding programs. Biol. Conserv..

[10] Wang YL, Binkowski FP, Doroshov SI (1985). Effect of temperature on early development of white and lake sturgeon *Acipenser transmontanus* and *A. fulvescens*. Environ. Biol. Fish..

[11] Hildebrand L (2016). Status of White Sturgeon (*Acipenser transmontanus* Richardson, 1863) throughout the species range, threats to survival, and prognosis for the future. J. Appl. Ichthyol..

[12] de Siqueira-Silva DH (2018). Biotechnology applied to fish reproduction: tools for conservation. Fish Physiol. Biochem..

[13] Jin YH, Robledo D, Hickey JM, McGrew MJ, Houston RD (2021). Surrogate broodstock to enhance biotechnology research and applications in aquaculture. Biotechnol. Adv..

[14] Rivers N, Daly J, Temple-Smith P (2020). New directions in assisted breeding techniques for fish conservation. Reprod. Fertil. Dev..

[15] Yoshizaki G, Yazawa R (2019). Application of surrogate broodstock technology in aquaculture. Fish. Sci..

[16] Pšenička M, Saito T, Yoshida M, Asturiano JF (2020). Chapter 16 specificity of germ cell technologies in sturgeons. Reproduction in Aquatic Animals: From Basic Biology to Aquaculture Technology.

[17] Okutsu T, Suzuki K, Takeuchi Y, Takeuchi T, Yoshizaki G (2006). Testicular germ cells can colonize sexually undifferentiated embryonic gonad and produce functional eggs in fish. Proc. Natl. Acad. Sci..

[18] Yoshizaki G (2010). Sexual plasticity of ovarian germ cells in rainbow trout. Development.

[19] Takeuchi Y, Yoshizaki G, Takeuchi T (2003). Generation of live fry from intraperitoneally transplanted primordial germ cells in rainbow trout. Biol. Reprod..

[20] Yazawa R (2010). Chub mackerel gonads support colonization, survival, and proliferation of intraperitoneally transplanted xenogenic germ cells. Biol. Reprod..

[21] Hamasaki M (2017). Production of tiger puffer *Takifugu rubripes* offspring from triploid grass puffer *Takifugu niphobles* parents. Mar. Biotechnol..

[22] Saito T, Goto-Kazeto R, Arai K, Yamaha E (2008). Xenogenesis in teleost fish through generation of germ-line chimeras by single primordial germ cell transplantation. Biol. Reprod..

[23] Farlora R (2014). Intraperitoneal germ cell transplantation in the Nile tilapia *Oreochromis niloticus*. Mar. Biotechnol..

[24] Morita T (2015). Functional sperm of the yellowtail (*Seriola quinqueradiata*) were produced in the small-bodied surrogate, jack mackerel (*Trachurus japonicus*). Mar. Biotechnol..

[25] Okutsu T, Shikina S, Kanno M, Takeuchi Y, Yoshizaki G (2007). Production of trout offspring from triploid salmon parents. Science.

[26] Pšenička M, Saito T, Linhartová Z, Gazo I (2015). Isolation and transplantation of sturgeon early-stage germ cells. Theriogenology.

[27] Takeuchi Y, Yoshizaki G, Takeuchi T (2004). Surrogate broodstock produces salmonids. Nature.

[28] Ye H (2017). Establishment of intraperitoneal germ cell transplantation for critically endangered Chinese sturgeon *Acipenser sinensis*. Theriogenology.

[29] Keyvanshokooh S, Gharaei A (2010). A review of sex determination and searches for sex-specific markers in sturgeon. Aquacult. Res..

[30] Takeuchi Y, Yazawa R, Yoshizaki G, Yoshida M, Asturiano JF (2020). Chapter 17 intraperitoneal germ cell transplantation technique in marine teleosts. Reproduction in aquatic animals: From basic biology to aquaculture technology.

[31] Menn, F.L., Benneteau-Pelissero, C. & Menn, R.L. An updated version of histological and ultrastructural studies of oogenesis in the Siberian sturgeon *Acipenser baerii*. in *The Siberian Sturgeon (Acipenser baerii, Brandt, 1869) Volume 1-Biology*, 279–305 (Springer, 2018).

[32] Nagai T, Yamaha E, Arai K (2001). Histological differentiation of primordial germ cells in zebrafish. Zool. Sci..

[33] Kuwana T, Fujimoto T (1983). Active locomotion of human primordial germ cells in vitro. Anat. Rec..

[34] Lubzens E, Young G, Bobe J, Cerdà J (2010). Oogenesis in teleosts: How fish eggs are formed. Gen. Comp. Endocrinol..

[35] Braat AK, Zandbergen T, Van De Water S, Goos HJT, Zivkovic D (1999). Characterization of zebrafish primordial germ cells: Morphology and early distribution of vasa RNA. Dev. Dyn..

[36] Zhang Z, Hill J, Holland M, Kurihara Y, Loveland KL (2008). Bovine Sertoli cells colonize and form tubules in murine hosts following transplantation and grafting procedures. J. Androl..

[37] Pšenička M, Saito T, Rodina M, Dzyuba B (2016). Cryopreservation of early stage Siberian sturgeon *Acipenser baerii* germ cells, comparison of whole tissue and dissociated cells. Cryobiology.

[38] Takeuchi Y (2018). Production of functionally sterile triploid Nibe croaker *Nibea mitsukurii* induced by cold-shock treatment with special emphasis on triploid aptitude as surrogate broodstock. Aquaculture.

[39] Havelka, M. Molecular aspects of interspecific hybridization of sturgeons related to polyploidy and in situ conservation. *PhD Diss. University of South Bohemia in Ceské Budejovice, Faculty of Fisheries and Protection of Waters* (2013).

[40] Baloch AR (2019). Dnd1 knockout in sturgeons by CRISPR/Cas9 generates germ cell free host for surrogate production. Animals.

[41] Linhartová Z (2015). Sterilization of sterlet *Acipenser ruthenus* by using knockdown agent, antisense morpholino oligonucleotide, against dead end gene. Theriogenology.

[42] Saito, T., Guralp, H., Iegorova, V., Rodina, M. & Psenicka, M. Elimination of primordial germ cells in sturgeon embryos by UV-irradiation. *Biol. Reprod. *(2018). 10.1093/biolre/ioy076 PMC6134207 29635315

[43] COSEWIC (2012). COSEWIC Assessment and Status Report on the White Sturgeon Acipenser transmontanus in Canada.

[44] Duke S (1999). Recovery plan for Kootenai River white sturgeon (*Acipenser transmontanus*). J. Appl. Ichthyol..

[45] Ireland SC, Beamesderfer R, Paragamian V, Wakkinen V, Siple J (2002). Success of hatchery-reared juvenile white sturgeon (*Acipenser transmontanus*) following release in the Kootenai River, Idaho, USA. J. Appl. Ichthyol..

[46] Thorstensen M, Bates P, Lepla K, Schreier A (2019). To breed or not to breed? Maintaining genetic diversity in white sturgeon supplementation programs. Conserv. Genet..

[47] Willis SC (2022). Population structure of white sturgeon (*Acipenser transmontanus*) in the Columbia River inferred from single-nucleotide polymorphisms. Diversity.

[48] Adams PB (2007). Population status of North American green sturgeon, *Acipenser medirostris*. Environ. Biol. Fish..

[49] Vick P (2021). Southern Distinct Population Segment of North American Green Sturgeon (Acipenser medirostris) 5-Year Review: Summary and Evaluation.

[50] Van Eenennaam JP (2001). Artificial spawning and larval rearing of Klamath River green sturgeon. Trans. Am. Fish. Soc..

[51] Underwood, W. & Anthony, R. *AVMA Guidelines for the Euthanasia of Animals: 2020 Edition*. (2020).

[52] Lacerda SMDSN, Costa GMJ, de França LR (2014). Biology and identity of fish spermatogonial stem cell. Gen. Comp. Endocrinol..

[53] Bradford MM (1976). A rapid and sensitive method for the quantitation of microgram quantities of protein utilizing the principle of protein-dye binding. Anal. Biochem..

[54] Yazawa R, Takeuchi Y, Morita T, Ishida M, Yoshizaki G (2013). The Pacific bluefin tuna (*Thunnus orientalis*) dead end gene is suitable as a specific molecular marker of type A spermatogonia. Mol. Reprod. Dev..

[55] Duangkaew R, Jangprai A, Ichida K, Yoshizaki G, Boonanuntanasarn S (2019). Characterization and expression of a vasa homolog in the gonads and primordial germ cells of the striped catfish (*Pangasianodon hypophthalmus*). Theriogenology.

[56] Saito T, Psenicka M (2015). Novel technique for visualizing primordial germ cells in sturgeons (*Acipenser ruthenus*, *A. gueldenstaedtii*, *A. baerii*, and *Huso huso*). Biol. Reprod..

[57] Kim J, Jung H, Yoon M (2015). VASA (DDX4) is a putative marker for spermatogonia, spermatocytes and round spermatids in stallions. Reprod. Domest. Anim..

[58] Castrillon DH, Quade BJ, Wang T, Quigley C, Crum CP (2000). The human VASA gene is specifically expressed in the germ cell lineage. Proc. Natl. Acad. Sci..

[59] Hartung O, Forbes MM, Marlow FL (2014). Zebrafish vasa is required for germ-cell differentiation and maintenance. Mol. Reprod. Dev..

[60] Davarinejad H (2015). Quantifications of Western Blots with ImageJ.

