# Supplementary material for: Germ cell recovery, cryopreservation and transplantation in the California white sturgeon, Acipenser transmontanus
Source: Sci Rep. 2023 Oct 6;13:16905. doi: 10.1038/s41598-023-44079-6 (PMC10558555; doi:10.1038/s41598-023-44079-6)
Supplement: Supplementary file 1 — Supplementary Figure S1. [file 41598_2023_44079_MOESM1_ESM.pdf]

Germ Cell Recovery, Cryopreservation and Transplantation in the California White Sturgeon, *Acipenser transmontanus*

Amie L. T. Romney, Danielle M. Myers, Fatima R. Martin, Tawny N. Scanlan, Stuart A. Meyers

Supplementary Information

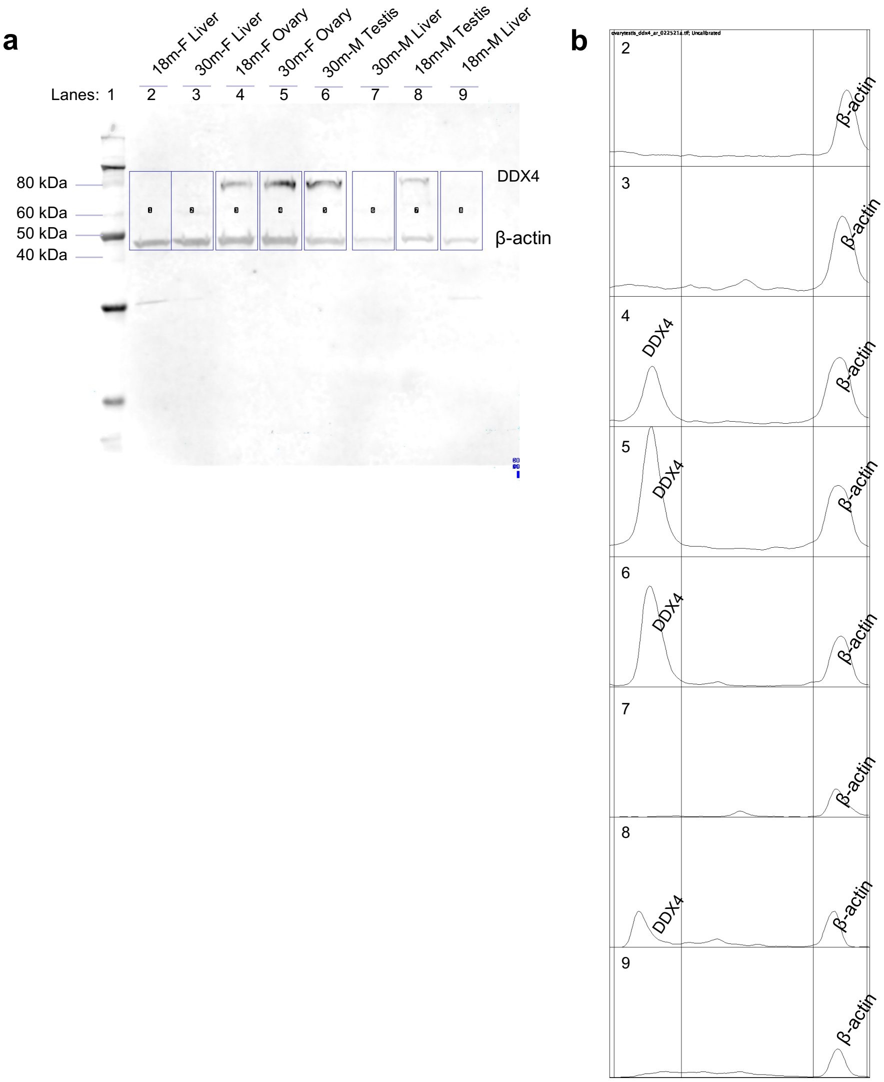

Supplemental Figure S1. Western blot analysis of germ cell marker, DEAD-box helicase 4 (DDX4) compared to the eukaryotic-cell control β-actin from gonadal protein. (a) In the full-length blot image, samples in lanes 2-9 belong to female and male (F = female and M = male) liver and gonad tissues (L = liver, O = ovary, and T = testis). Bands can be detected alongside the Novex Sharp prestained ladder (lane 1) consisting of 12 colored bands 3.5 - 260 kDa. Single bands in all protein samples at approximately 45 kDa are predicted as β-actin and the samples with the single band at approximately 75 kDa are predicted as Anti-DDX4 antibody staining. (b) Band pixel density analysis (densitometry) in sample lanes (2-9) using ImageJ according to Davarinejad[60]. Histograms represent the lane position (pixel; x-axis) by intensity (band area).
